# Practical Synthesis of Chalcone Derivatives and Their Biological Activities

**DOI:** 10.3390/molecules22111872

**Published:** 2017-11-01

**Authors:** Jae-Chul Jung, Yongnam Lee, Dongguk Min, Mankil Jung, Seikwan Oh

**Affiliations:** 1 Department of Molecular Medicine, School of Medicine, Ewha Womans University, Seoul 07985, Korea; jcjung@novarex.co.kr; 2 Department of Chemistry, Yonsei University, Seoul 03722, Korea; nami0209@naver.com (Y.L.); alsdog@yonsei.ac.kr (D.M.)

**Keywords:** chalcones, condensation reaction, free radical scavenging, NO generation, neurotoxicity, molecular modelling

## Abstract

Practical synthesis and biological activities of 4-hydroxy-3-methoxy-2-propene derivatives are described. The novel chalcone derivatives were prepared by acid catalysed one-step condensation of 1,3- or 1,4-diacetylbenzene and 1,3,5-triacetylbenzene with 4-hydroxy-3-methoxybenzaldehyde. They were then evaluated for free radical scavenging activity, suppression of lipopolysaccharides (LPS)-induced NO generation, and anti-excitotoxicity in vitro. It was found that all compounds showed good effects for 2,2-diphenyl-1-picrylhydrazyl (DPPH) free radical scavenging, LPS-induced NO generation, and anti-neurotoxicity. Compounds **6** and **7** were potent suppressor of NO generation with the concentration range 10 µM and especially compound **8** showed very potent anti-inflammatory activity with 1 µM. In addition, the di- and tri-acetylbenzyl derivatives **6**, **7**, and **8** showed enhanced anti-neurotoxicity activity in cultured cortical neurons. Molecular modelling studies to investigate the chemical structural characteristics required for the enhanced biological activities interestingly revealed that compound **8** has the smallest highest occupied molecular orbital-lowest energy unoccupied molecular orbital (HOMO-LUMO) gap, which signifies easy electron and radical transfer between HOMO and LUMO in model studies.

## 1. Introduction

**Figure 1:**
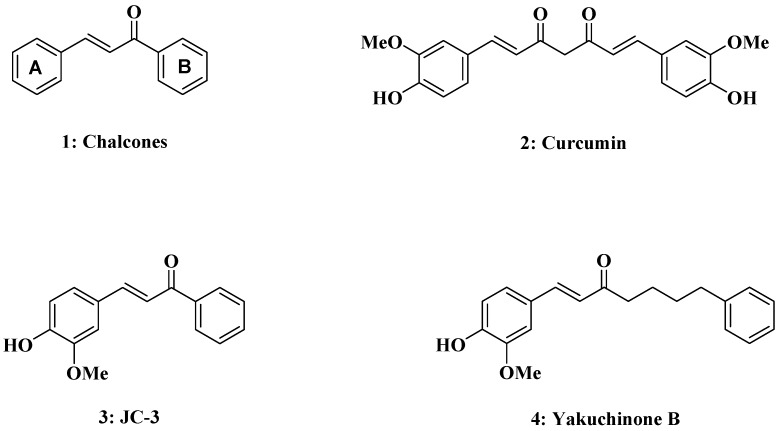
Structures of chalcone **1**, curcumin **2**, JC-3 **3**, and yakuchinone B **4**.

Most of the chalcone moieties have evoked a great deal of interest due to their biological properties and characteristic conjugated molecular architecture. Chalcones have been considered derivatives of the 1,3-diaryl-2-propene-1-one parent compound composed of two phenolic rings, referred to as the A and B rings. Many of them possess important pharmacological properties, such as analgesic [1], arthritis [2], anti-inflammatory [3], anti-pyretic [4], anti-bacterial [5], anti-viral [6,7], and anti-cancer [8,9] effects. They were also potentially useful for many industrial products and phytochemical applications, including food sciences. Nowadays, a number of comparative pharmacological investigations of the chalcones have showed good antioxidant activity with low side effects [10,11,12,13] (Figure 1). Especially, curcumin and its related enones, such as yakuchinones A and B, inhibited the activation of the prosurvival transcription factor nuclear factor-kβ (NF-kβ) and up-regulation of cyclooxygenase-2 (COX-2). The chemical synthesis, quantitative structural modification, and a wide variety of biological activities of chalcones were reported in many studies [14,15,16].

Naturally occurring chalcones derived from general foods are phloretin and its glucoside phloridzin chalconaringenin, and arbutin. Most of the studies to date, regarding the synthetic approaches of chalcone derivatives [17,18,19,20,21],were reported by the organic and medicinal chemists through the formation of the 1,4-enones using acid- or base-catalysed condensation reactions of aldehyde and aryl methyl ketones in alcoholic solvents with variable yields.

An interesting biological report of chalcone derivatives described the potential antioxidant activities of conjugated phenolic enones by the Vander Jagt group [22]. The Oh group described the neuroprotective effects of benzylideneacetophenone derivatives on excitotoxicity and inflammation via the phosphorylated janus tyrosine kinase 2/phosphorylated signal transducer and activator of transcription 3 and mitogen-activated protein K pathways, and compound **6** was more potent than compound **3** in the aspect of proteasome inhibition, which induced apoptosis significantly in the prostate cancer cells [23,24]. The Ryu group demonstrated that the optimal length of linker between aryl groups played an important role for the biological activity, and the Di Pietro group showed that potent bis-chalcone inhibitors were identified, the efficiency depending on both position of the central ketone groups and the number and positions of lateral methoxy substituents in breast cancer resistance protein inhibition [25,26].

In our previous study of 1,3-diaryl-2-propenones, we have developed a simple synthesis via the Grignard reaction of aldehyde and oxidation of secondary alcohols [27]. We also reported the one-step synthesis of benzylideneacetophenones and the evaluation of their biological activities [28]. We are continuously interested in the chemistry of chalcone derivatives, in part for their potential use as scaffolds in medicinal chemistry. We wish to report herein the simple synthesis and biological activities of dimer or trimer of 4-hydroxy-3-methoxy-2-propene derivatives **6**–**8** for free radical scavenging, suppression of lipopolycahharide (LPS)-induced NO generation in vitro, and anti-excitotoxicity, which were prepared by 4-hydroxy-3-methoxybenzaldehyde and diacetophenone or triacetophenone via acid catalysed one-pot condensation reaction in good yields. Biological activities of their derivatives for suppression of LPS-induced NO generation in vitro suggests that they can be possible anti-inflammatory lead compounds. Furthermore, molecular modelling studies were reported to investigate the chemical structural characteristics required for the biological activities and schematizes a delocalized form of electron density. These efforts might be help to develop and design more potent novel chalcones with neuroprotective effects.

## 2. Results and Discussion

### 2.1. Chemistry

Since chalcone synthesis has been achieved by employing the Suzuki reaction, the base-catalysed condensation has been accomplished with good yields [29]. In a model study, we investigated the reactivity of several bases, such as lithium hydroxide (LiOH), sodium hydroxide (NaOH), potassium hydroxide (KOH), magnesium hydroxide [Mg(OH)_2_], and barium hydroxide [Ba(OH)_2_]. Isovanillin was treated with 3-fluoro-4-methoxy acetophenone in methanol in the presence of two equivalents of bases to give (*E*)-1-(3-fluoro-4-methoxyphenyl)-3-(3-hydroxy-4-methoxyphenyl)-prop-2-en-1-one. An excellent result of the desired product was obtained using LiOH in methanol at room temperature for 148 h in 90% yield. 

Practically, most of the other bases showed good yields, while the reaction with Mg(OH)_2_ failed to afford 1,4-enones. Lithium hydroxide proved to be the superior base and consistently gave higher yields than the other bases. It seems probable that the effectiveness of lithium hydroxide can be explained in part by a lithium chelating effect. Based on these results, we modified to generate our desired dimer or trimer types of chalcone derivatives. Interestingly, the base for catalysing the Claisen-Schmidt condensation of 4-hydroxy-3-methoxybenzaldehyde and 1,3-diacetylbenzene, 1,4-diacetylbenzene or 1,3,5-triacetylbenzene is unsatisfied. 

To establish generality, the acid-catalysed condensation reaction of aldehyde with 1,3-diacetylbenzene or 1,4-diacetylbenzene and 1,3,5-triacetylbenzene under mild reaction conditions was carried out in the presence of various acids, such as AcOH, *c*-HCl, *c*-H_2_SO_4_, H_3_PO_4_, BF_3_-EtO_2_, AlCl_3_, and Montmorillonite K 10. The reasonable result was obtained in the case of the use of stoichiometric amounts of *c*-sulphuric acid in ethanol. The reaction was monitored by thin-layer chromatography (TLC) and gas chromatography- mass spectrometry (GC-MS) and easy to perform without any elaborative work-up. 

**Scheme 1:**
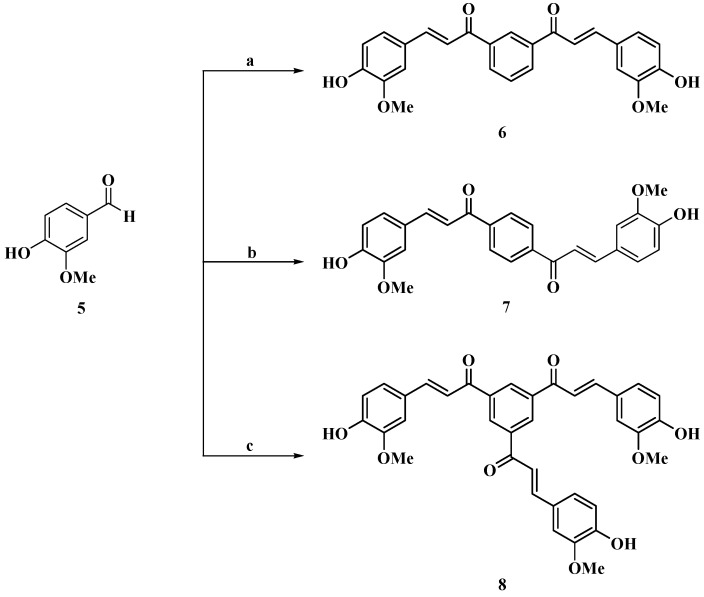
Reagents and conditions: (**a**) 1,3-diacetylbenzene, *c*-H_2_SO_4_, EtOH, reflux 3 h; (**b**) 1,4-diacetylbenzene, *c*-H_2_SO_4_, EtOH, reflux 3 h; and (**c**) 1,3,5-triacetylbenzene, *c*-H_2_SO_4_, EtOH, reflux 3 h.

In the continuous of our medicinal program dealing with the development of new 1,3-diaryl-2-propen-1-one derivatives, we have introduced dimer and trimer types of the benzylideneacetophenone backbone in order to develop antioxidant agents and anti-excitotoxic compounds. 4-Hydroxy-3-methoxy-2-propene derivatives **6**–**8** were prepared from commercially available 4-hydroxy-3-methoxybenzaldehyde 5 and 1,3-diacetylbenzene, 1,4-diacetylbenzene or 1,3,5-triacetylbenzene in the presence of stoichiometric amounts of *c*-sulphuric acid in ethanol to give 3-(4-hydroxy-3-methoxy-phenyl)-1-{3-[3-(4-hydroxy-3-methoxy-phenyl)-acryloyl]-phenyl}-propenone, 3-(4-hydroxy-3-methoxy-phenyl)-1-{4-[3-(4-hydroxy-3-methoxy-phenyl)-acryloyl]-phenyl}-propenone, and 1-{3,5-bis-[3-(4-hydroxy-3-methoxy-phenyl)-acryloyl]-phenyl}-3-(4-hydroxy-3-methoxy-phenyl)-propenone in 23%, 35%, and 73% yields, respectively (Scheme 1).

### 2.2. Molecular Modeling 

**Figure 2:**
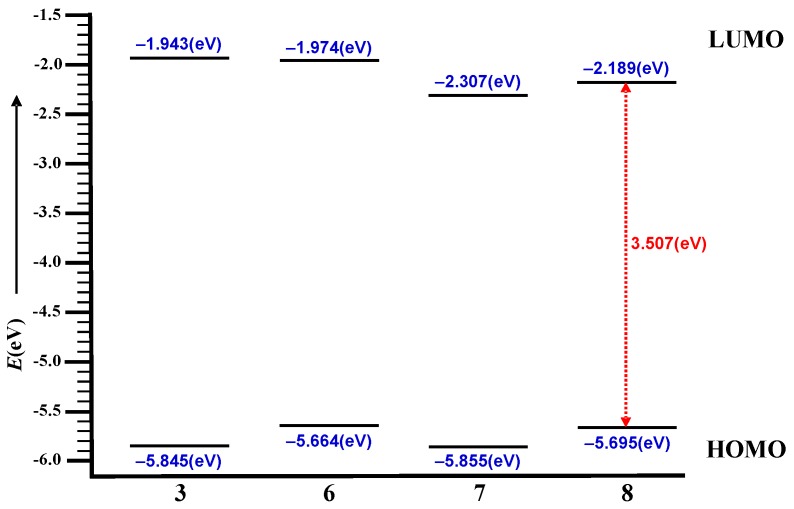
HOMO-LUMO gaps of chalcone derivatives **3** and **6**–**8**.

**Figure 3:**
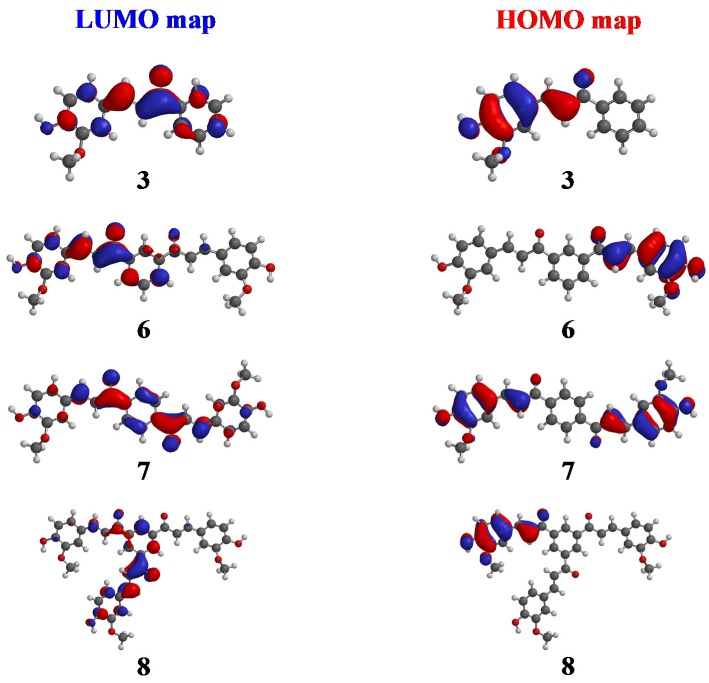
LUMO and HOMO maps of chalcones derivatives **3** and **6**–**8**.

The main objectives of the molecular modelling studies were to investigate the effects of monomer, dimmer, and trimer on free radical scavenging, cell viability, and LPS-induced nitric oxide generation in chalcone derivatives **3**, and **6**–**8** to investigate their neuroprotective effects. The results of the molecular modelling studies of chalcone derivatives **3** and **6**–**8** are as shown in Table 1. Although diverse compounds were not used in this modelling study, a significant result was obtained. The highest-occupied molecular orbital (HOMO) and the lowest-unoccupied molecular orbital (LUMO) energies ranged between −5.855 and −5.664 and −2.307 and −1.943 eV, respectively. Especially, compound **8** had the smallest HOMO-LUMO gap (3.507 eV), which signifies rapid electron and radical transfer between HOMO and LUMO (Figure 2). This could be one of the reasons that compound **8** showed good free radical scavenging activity. This finding reveals a close relationship to the electro density of compound **8** based on resonance effect, which is represented in Figure 3. On the basis of these results, we could expect that the HOMO-LUMO gap could be considered to be important parameters for choosing anti-neurotoxic compounds among the evaluated chalcone derivatives.

### 2.3. Biological Evaluation

#### 2.3.1. Radical Scavenging Activity

**Figure 4:**
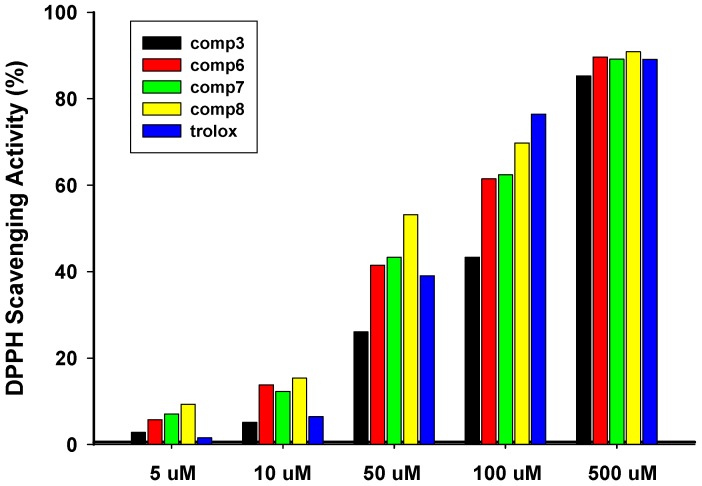
Rate of scavenging 2,2-diphenyl-1-picrylhydrazyl (DPPH) radical of analogues **3** and **6**–**8**.

2,2-Diphenyl-1-picrylhydrazyl (DPPH) radicals are representative method for the preliminary screening of compounds capable of scavenging activated oxygen species since they are much more stable and easier to handle than oxygen free radicals. The radical scavenging activity of the 1,3-diphenyl-2-propen-1-ones, **3** and **6**–**8** were evaluated by the known method [30,31] and the results are summarized in Figure 4. All these compounds exhibited free radical scavenging ability at concentrations of 5 µM, 10 µM, 50 µM, 100 µM, and 100 µM as compared with control material, respectively. Interestingly prepared compounds **6**–**8** showed higher DPPH radical scavenging activity than that of standard compound, trolox (6-hydroxy-2,5,7,8-tetramethylchroman-2-carboxylic acid) [32]. Additionally, compounds **6**–**8** showed good DPPH radical scavenging activity compared with trolox at the concentration of 50 and 100 µM. Estimation of the structural characteristics of prepared chalcone derivatives revealed that the major feature is composed according to monomers, dimers, and trimers based on the 1,4-phenethyldion conjugated skeleton. Compound **8** exhibited the most potent radical scavenging activity among these analogues. This finding suggests that the trimer group significantly enhanced radical scavenging and antioxidant activity due to internal electronic effect and/or favourable binding to the active sites.

#### 2.3.2. Inhibition of NO Generation

**Figure 5:**
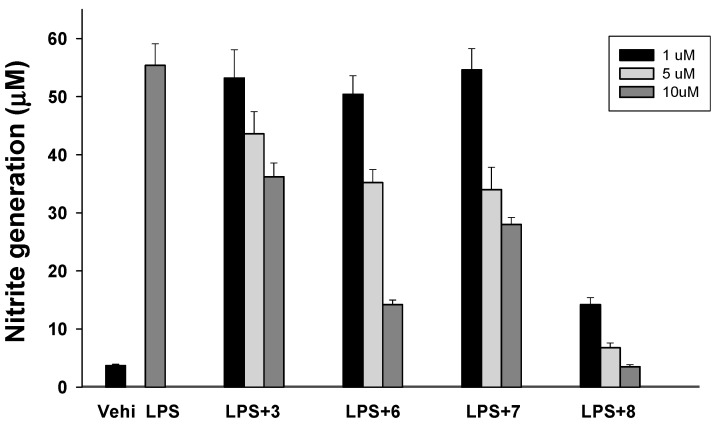
Suppression of NO production in lipopolysaccharide (LPS–treated BV2 cells. The cells were treated with 1 µg/mL of LPS only or LPS plus different concentration (1, 5 and 10 µM) of compounds **3** and **6**–**8** at 37 °C for 24 h. At the end of incubation, 50 µL of the medium was taken to measure the nitrite production. All values represent the mean ± Standard Error (SE) of three-independent experiments performed in triplicate.

The in vitro suppression of NO production of compounds **3** and **6**–**8** were evaluated in LPS–treated microglia cells, and the results are summarized in Figure 5. Interestingly, dimer moiety compound **6** and **7** showed higher inhibitory activity of NO generation than that of the single moiety **3** at 5 and 10 µM. It was found that all compounds showed suppression of NO generation on microglia cells, with the most potent inhibition trimer compound **8**, even at a concentration of 1 µM. This result implied that the methoxyl and *para*-position methoxyl group at the benzene ring enhanced to delocalize for the electron density based on resonance effect. Compounds with dimer and timer methoxyl and hydroxyl groups generally showed increased suppression of NO production.

#### 2.3.3. Neuroprotective Activity: Inhibition of Glutamate-Induced Neurotoxicity

**Figure 6:**
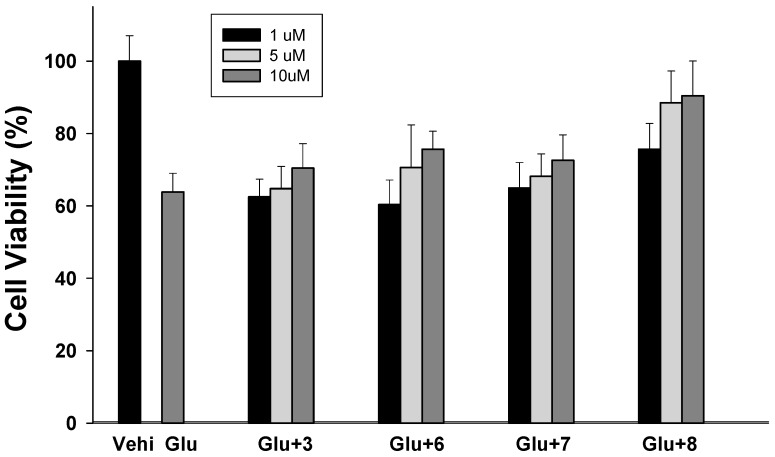
Inhibition of glutamate-induced neurotoxicity in cultured cortical neurons. Glutamate alone or with (60 µM), compounds **3** and **6**–**8** were applied for 24 h at 37 °C. After incubation of neurons with WST-1 for 2 h and quantified spectrophotometrically. All values represent the mean ± SE of three-independent experiments performed in triplicate.

We have examined the neuroprotective effects of **3** and **6**–**8** on the inhibition of glutamate-induced neurotoxicity in cultured cortical neurons. Most of the chalcone compound showed good anti–excitotoxicity on the concentration range over 10 µM as shown in Figure 6. Compound **8** exhibited the most potent activity among these analogues. The increasing efficacy of hydroxyl moieties **6**–**8** is presumably due to the *para*-position, which has increased the electron density of the hydroxyl group and lowered the oxygen–hydrogen bonding energy.

## 3. Materials and Methods

### 3.1. Synthesis

All commercial reagents and solvents were used as received without further purification unless specified. The reactions were monitored and the *R_f_* values determined using TLC with Merck silica gel 60, F-254 pre-coated plates (0.25-mm thickness) (Merck, Frankfurt, Germany). Spots on the TLC plates were visualized using ultraviolet light (254 nm) (Spectroline, New York, NY, USA) and a basic potassium permanganate solution or cerium sulfate/ammonium dimolybdate/sulfuric acid solution in house, followed by heating on a heat-gun, PHG 630 DCE (BOSCH, Gerlingen, Germany). ^1^H-Nuclear Magnetic Resonance (NMR) spectra were recorded on Bruker DPX-250 (Bruker Optics, Billerica, MA, USA) spectrometers. Proton chemical shifts are reported in ppm (δ) relative to internal tetramethylsilane (TMS, δ 0.00) or with the solvent reference relative to TMS as the internal standard (CDCl_3_, δ 7.26 ppm; *d*_4_-CD_3_OD, δ 3.31 ppm, *d*_6_-DMSO, δ 2.50 ppm). Data are reported as follows: chemical shift multiplicity [singlet (s), doublet (d), triplet (t), quartet (q), and multiplet (m)], coupling constants [Hz], integration. ^13^C-NMR spectra were recorded on Bruker DPX-250 (63 MHz) spectrometers with complete proton decoupling. Carbon chemical shifts are reported in ppm (δ) relative to TMS with the respective solvent resonance as the internal standard (CDCl_3_, δ 77.0 ppm; *d*_4_-CD_3_OD, δ 49.0 ppm, *d*_6_-DMSO, δ 39.5 ppm). Infrared (IR) spectra were recorded on a Nicolet Model Impact FT-IR 400 spectrometer (Thermo Fisher Scientific, Waltham, MA, USA). Data are reported in wave numbers (cm^−1^). Mass spectra were recorded on a Matrix Assisted Laser Desorption/Ionization-Time-of-Flight Mass Spectrometry (MALDI-TOF) Voyager-DE STR (Applied Biosystems 4700 proteomics analyser spectrometer, Palo Alto, CA, USA) with a α-cyano-4-hydroxycinnamic acid (α-CHCA) matrix. High-Resolution Mass Spectrometry (HRMS) was recorded on a LTQ Orbitrap Velos Liquid Chromatography Mass Spectrometry (LC-MS) (Thermo Fisher Scientific, Waltham, MA, USA). Electrospray ionization (ESI)-LC-MS was recorded on a Waters ZQ 4000 LC-MS spectrometer (Waters Corporation, Milford, MA, USA). Purified human 20S proteasome was purchased from Enzo Life Sciences (Plymouth Meeting, PA, USA). Fluorogenic peptide substrates Suc-LLVY-AMC was obtained from Sigma-Aldrich (St. Louis, MO, USA).

*(E)-1-{3-[(E)-3-(4-Hydroxy-3-methoxyphenyl)acryloyl]phenyl}-3-(4-hydroxy-3-methoxyphenyl)-2-propenone* (**6**). To a stirred solution of 1,3-diacetylbenzene (0.24 g, 1.48 mmol) and *c*-H_2_SO_4_ (0.15 g, 1.48 mmol) in ethanol (10 mL) was refluxed for 30 min and then a solution of vanillin (0.45 g, 2.96mmol) in ethanol (10 mL) was added to the reaction mixture. The resulting solution was refluxed for 24 h. The reaction mixture was cooled to room temperature and concentrated under reduced pressure. The residue was purified by flash column chromatography (silica gel, 30% ethyl acetate/hexanes) to give pure product **6** as a brown solid (0.3 g, 23%). R*_f_*= 0.28 (dichloromethane/methanol = 50:1, *v*/*v*); m.p. 165–168 °C; IR ν_max_ (CHCl_3_, KBr) 3394, 1655, 1585, 1512, 1463, 1450, 1429, 1270, 1204, 1178, 1124, 1031, 980, 845, 755 cm^−1^; ^1^H-NMR (250 MHz, CDCl_3_) δ 8.62 (s, 1 H), 8.20 (dd, *J* = 7.58, 1.58 Hz, 2H), 7.80 (d, *J* = 15.48 Hz, 2H), 7.62 (t, *J* = 7.74 Hz, 1H), 7.42 (d, *J* = 15.48 Hz, 2H), 7.23 (d, *J* = 1.90 Hz, 1H), 7.20 (d, *J* = 1.90 Hz, 1H), 7.15 (d, *J* = 1.58 Hz, 2H), 6.96 (d, *J* = 8.21 Hz, 2H), 3.94 (s, 6H); ^13^C-NMR (63 MHz, CDCl_3_) δ190.06, 148.80, 147.04, 146.26, 138.91, 132.31, 129.05, 128.28, 127.31, 123.97, 119.23, 115.09, 110.20, 56.16; MALDI-TOF [M + H] 431.0972; HRMS calcd. for C_26_H_22_O_6_: 430.1416 [M]^+^, found: 430.1421. 

*(E)-1-{4-[(E)-3-(4-Hydroxy-3-methoxyphenyl)acryloyl]phenyl}-3-(4-hydroxy-3-methoxyphenyl)-2-propenone* (**7**). To a stirred solution of 1,4-diacetylbenzene (0.053 g, 0.33 mmol) and *c*-H_2_SO_4_ (0.032 g, 0.33 mmol) in ethanol (5 mL) was refluxed for 30 min and a solution of vanillin (0.1 g, 0.66 mmol) in ethanol (5 mL) was added to the reaction mixture. The resulting mixture was refluxed for 12 h. The reaction mixture was cooled to room temperature. The solid was filtered and washed with ethanol and ether to give pure product **7** as a brown solid (0.05 g, 35%). R*_f_* = 0.6 (dichloromethane/methanol = 9:1, *v*/*v*); m.p. 228–230 °C; IR ν_max_ (acetone, KBr) 3423, 2926, 2850, 1728, 1649, 1582, 1514, 1430, 1384, 1272, 1161, 1126, 1092, 1030, 815, cm^−1^; ^1^H-NMR (250 MHz, acetone-*d*_6_) δ 8.22 (s, 4H), 7.74–7.77 (m, 4H), 7.53 (s, 2H), 7.34 (dd, *J* = 2.0, 8.2 Hz, 2H), 6.92 (d, *J* = 8.2 Hz, 2H), 3.94 (s, 6H); ^13^C-NMR (63 MHz, acetone-*d*_6_) δ 189.9, 150.8, 149.0, 146.5, 129.5, 128.1, 124.9, 120.2, 116.4, 112.4, 109.8, 56.6; LC-MS (ESI) *m*/*z* 453 [M + Na]^+^; MALDI-TOF [M + H] 431.1430; HRMS calcd. for C_26_H_22_O_6_: 430.1416 [M]^+^, found: 430.1432. 

*(E)-1-{3,5-Bis[(E)-3-(4-hydroxy-3-methoxyphenyl)acryloyl]phenyl}-3-(4-hydroxy-3-methoxyphenyl)-2-propenone* (**8**). To a stirred solution of 1,3,5-triacetylbenzene (0.045 g, 0.22 mmol) and *c*-H_2_SO_4_ (0.022 g, 0.22 mmol) in ethanol (5 mL) was refluxed for 30 min and then a solution of vanillin (0.1 g, 0.66 mmol) in ethanol (5 mL) was added to the reaction mixture. The resulting mixture was refluxed for 3 h and cooled to room temperature. The solid was filtered and washed with ethanol and ether to give pure product **8** as a brown solid (0.097 g, 73%). R*_f_* = 0.6 (dichloromethane/methanol = 9:1, *v*/*v*); m.p. 255–256 °C; IR_max_ (acetone, KBr) 3435, 3062, 2933, 1649, 1591, 1514, 1429, 1384, 1269, 1161, 1124, 1029, 810 cm^−1^; ^1^H-NMR (250 MHz, acetone-*d*_6_) δ8.89 (s, 3H), 7.85–7.88 (m, 6H), 7.58 (s, 3H), 7.29 (d, *J* = 8.3 Hz, 3H), 6.92 (d, *J* = 8.2 Hz, 3H), 3.94 (s, 9H); ^13^C-NMR (100 MHz, acetone-*d*_6_) δ 189.4, 150.9, 148.9, 147.0, 140.6, 132.1, 128.1, 125.2, 120.0, 116.4, 112.5, 56.6; LC-MS (ESI+) *m*/*z* 629 [M + Na]. MALDI-TOF [M + H] 607.1219; HRMS calcd. for C_36_H_30_O_9_: 606.1890 [M]^+^, found: 606.1885. 

### 3.2. Molecular Modeling

The lower energy conformers for each compound, compounds **3** and **6**–**8** were searched with the semi-empirical AM1 method [33]. The lower energy conformers were submitted to a geometry optimization and energy calculations by density functional theories (DFT) model calculation at the B3LYP 6-31G** level [34]. The HOMO and LUMO values of the selected conformers were also calculated. All calculations and graphical representations were performed by using the SPARTAN 06 for Windows software package (SPARTAN 06 for Windows, Wavefuction Inc., Irvine, CA, USA) [35].

### 3.3. Biology-Measurement of Cell Viability

Cortical neuronal cell number and viability were assessed by using the reagent water soluble tetrazolium-1 (WST-1) (Roche, Indianapolis, IN). This colorimetric assay measures the metabolic activity of viable cells based on cleavage of the tetrazolium salt WST-1 substrate 4-[3-(4-iodophenyl)-2-(4-nitrophenyl)-2*H*-5-tetrazolio]-1,3-benzene disulfonate into formazan by mitochondria dehydrogenase in live cells. This was followed by incubation with WST-1 reagent at a dilution of 1:10 in the original conditioned media at 37 °C for 2 h. After thorough shaking, the formazan produced by the metabolically active cells in each sample was measured at a wavelength of 450 nm and a reference wavelength of 650 nm. Absorbance readings were normalized against control wells with untreated cells. Neuronal death was analysed 24 h later, and the percentage of neurons undergoing actual neuronal death was normalized to the mean value that was found after a 24 h exposure to 300 µM *N*-methyl-d-aspartate (NMDA) (defined as 0) or a sham control (defined as 100).

## 4. Conclusions

Simple synthesis involving one-step aldol condensation and biological properties of 4-hydroxy-3-methoxyphenyl-2-propenes **6**–**8** have been described. A simple synthetic strategy was established with an aldol reaction of 4-hydroxy-3-methoxybenzaldehyde and acetophenones in the presence of acidic media to generate novel chalcones **6**–**8**. These analogues have been contributed to form the stable phenoxy radical based on delocalizing electron movement and intramolecular hydrogen bonding. It was found that all compounds showed good effects for DPPH free radical scavenging, LPS-induced NO generation, and anti-neurotoxicity. Compounds **6** and **7** were potent suppressor of NO generation with the concentration range 10 µM and especially compound **8** showed very potent anti-inflammatory activity with 1 µM. The di- and tri-acetylbenzyl derivatives **6**, **7**, and **8** showed enhanced anti-neurotoxicity activity in cultured cortical neurons. Furthermore, the HOMO-LUMO gap could be considered to be potential explanations for the anti-neurotoxic effects of chalcone derivatives.

## Figures and Tables

**Table 1:** Results of the molecular modelling study for benzylideneacetophenone derivatives.

Compounds	Energy	E. HOMO ^a^	E. LUMO ^b^	Δ*E* ^c^
	(au)	(eV)	(eV)	(eV)
**3**	−843.796	−5.845	−1.943	3.901
**6**	−1455.342	−5.664	−1.974	3.690
**7**	−1455.334	−5.855	−2.307	3.548
**8**	−2066.890	−5.695	−2.189	3.507

^a^ Energy of highest-occupied molecular orbital. ^b^ Energy of lowest-unoccupied molecular orbital. ^c^ Δ*E* represents the orbital energy difference. HOMO: the highest-occupied molecular orbital; LUMO: the lowest-unoccupied molecular orbital.
